# A Systematic Review of Fibrin Glue as an Ideal Treatment for the Pilonidal Disease

**DOI:** 10.7759/cureus.16831

**Published:** 2021-08-02

**Authors:** Myat Win, Terry R Went, Sheila W Ruo, Amudhan Kannan, Jerry Lorren Dominic, Waleed Sultan, Ketan Kantamaneni, Vijaya lakshmi Yanamala, Anjli Tara, Abeer O Elshaikh

**Affiliations:** 1 General Surgery, Nottingham University Hospitals NHS Trust, Nottingham, GBR; 2 Surgery, California Institute of Behavioral Neurosciences & Psychology, Fairfield, USA; 3 Research, California Institute of Behavioral Neurosciences & Psychology, Fairfield, USA; 4 Surgery, Jawaharlal Institute of Postgraduate Medical Education and Research, Puducherry, IND; 5 General Surgery and Orthopaedic Surgery, Cornerstone Regional Hospital, Edinburg, USA; 6 General Surgery, Stony Brook Southampton Hospital, New York, USA; 7 Surgery, LaSante Health Center, Brooklyn, USA; 8 Medicine, Beni Suef University Faculty of Medicine, Beni Suef, EGY; 9 Neurology, California Institute of Behavioral Neurosciences & Psychology, Fairfield, USA; 10 Surgery, Halifax Health Medical Center, Daytona Beach, USA; 11 Surgery, Dr.Pinnamaneni Siddhartha Institute Of Medical Sciences And Research Foundation, Gannavaram, IND; 12 General Surgery, Liaquat University of Medical and Health Sciences, Jamshoro, PAK; 13 Internal Medicine, California Institute of Behavioral Neurosciences & Psychology, Fairfield, USA

**Keywords:** pilonidal sinus treatment, postoperative recovery, fibrin glue, outcome measure, adolescent and young adults

## Abstract

Pilonidal sinus is an acquired condition caused by irritation to the hair follicles at the natal cleft, presenting with an abscess or chronic infection. It is prevalent in young adults affecting their productive lifestyle with morbidities. There are varieties of treatment options; however, there is no consensus yet for the ideal procedure. Less invasive procedures have evolved to replace the traditional surgical techniques, which cannot significantly reduce the risks of recurrence and wound complications despite extensive surgeries. We aimed to assess the effect of fibrin glue as a primary treatment after cleaning the sinus in pilonidal sinus disease. We searched for articles from PubMed®, Ovid MEDLINE®, Ovid EMBASE®, and Cochrane CENTRAL. Six studies that included 336 patients in total were analyzed. Fibrin glue treatment in these studies reported a quicker return to normal activities postoperatively, a low rate of infection, and an acceptable rate of recurrence. Thus, fibrin glue seems beneficial in the management of pilonidal disease. However, further high-quality studies are essential to support and confirm this evidence. Future research should also evaluate its cost and implications in the ambulatory service.

## Introduction and background

The pilonidal term, firstly introduced in 1833, is derived from a combination of Latin words which are *pilus *(hair) and *nidus *(nest). Although there is no precise etiopathogenesis for this disease, the hypothesis is related to the recurrent erosion of hair follicles at the natal cleft [1]. A nest of loose hair leads to an inflammatory process and infection at the sacrococcygeal area. As a result, patients will suffer from pain, discharge, and itchiness around the natal cleft [2]. The risk factors are obesity, hirsutism, deep gluteal cleft, sitting for several hours per day, and family history [2, 3]. It can lead to various presentations such as acute abscess, chronic sinus, simple cyst, or complex condition with multiple recurrences [4]. 

It commonly affects young adults, especially males whose prevalence is two to three times higher than females [3]. It can cause socio-economic issues due to the high rate of recurrence affecting daily activities, absence from work, and school [5]. We can see the health burden of pilonidal disease with the incidence of nearly 25 per 100,000, affecting 0.7% of the population [2]. 

There are various types of management for pilonidal disease ranging from minimally invasive techniques to extensive surgical methods [6]. Less traumatic procedures such as laser epilation, pit picking, endoscopic pilonidal sinus treatment, video-assisted ablation of pilonidal sinus, the application of fibrin glue, thrombin gelatin, and phenol are being used in the clinical practice in addition to traditional surgical methods such as excision of sinus tract followed by primary or secondary closure and flap techniques [4]. However, there is no consensus yet for the ideal approach. The method which results in a speedy recovery, fewer complications, and minimal recurrence would be the perfect treatment [6].

**Figure 1:**
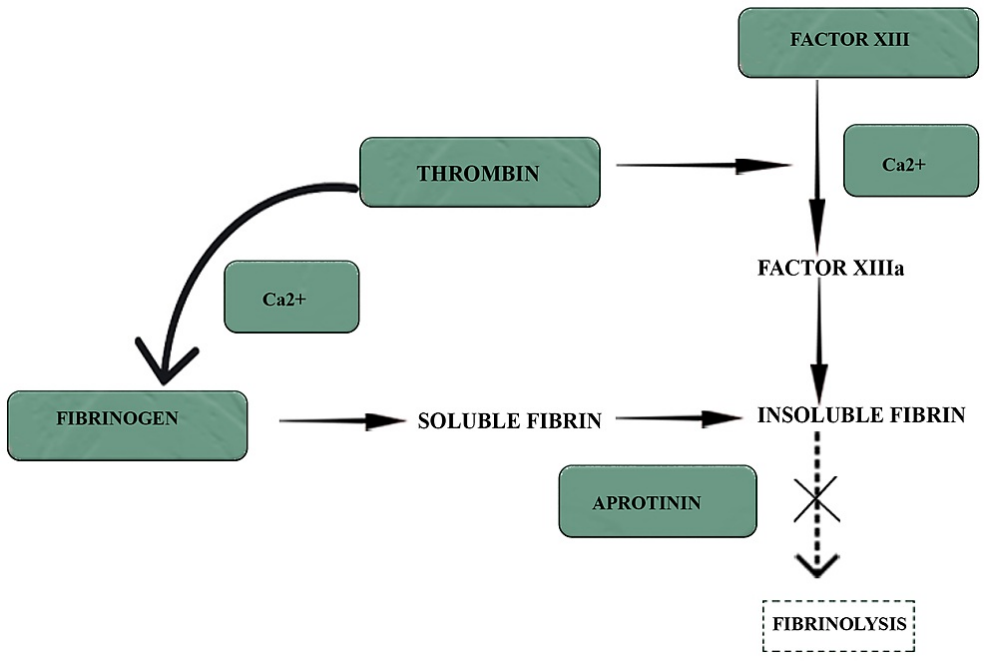
Mechanism of action of components included in fibrin glue

The content of fibrin glue includes a mixture of fibrinogen, thrombin, factor XIII, calcium, and aprotinin [7]. Thrombin activates the coagulation pathway by converting fibrinogen to fibrin, leading to fibrin clot formation. Factor XIII and calcium also play a role in the facilitation of this pathway. Aprotinin inhibits fibrinolysis, thereby maintaining the construction of stable fibrin [7]. This mechanism of action of ingredients in fibrin glue is shown in Figure 1. Its components were highlighted in green in this figure.

**Figure 2:**
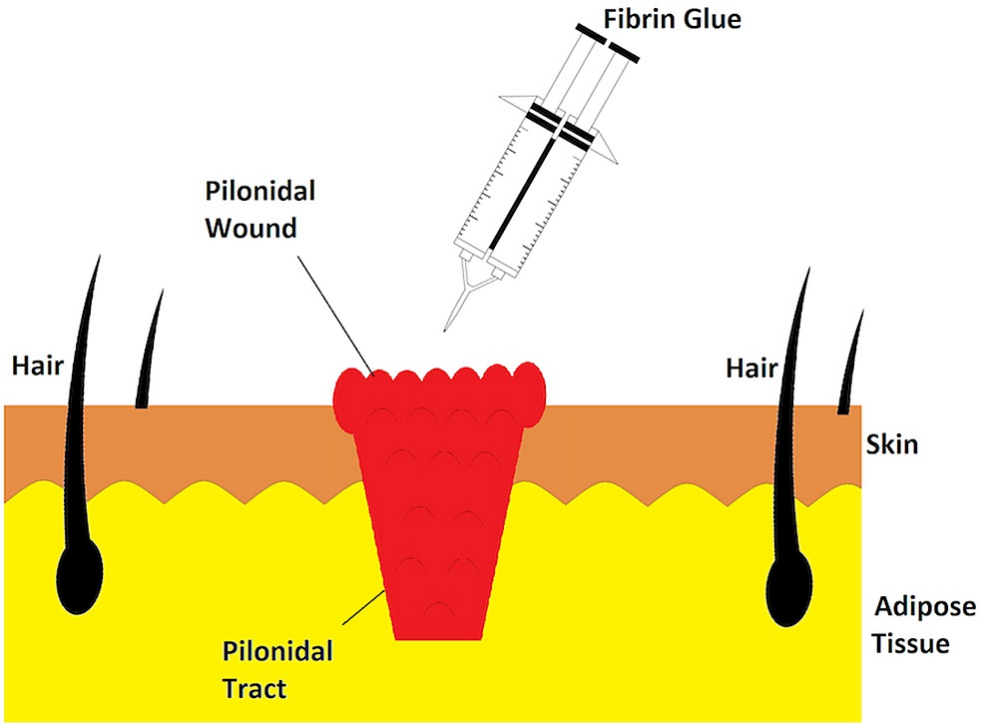
Illustration of fibrin glue application into the pilonidal wound

Fibrin sealant also facilitates wound healing with the effect of hemostasis followed by the invasion of macrophage, angiogenesis, and collagen deposition [8]. The therapeutic impact of fibrin glue to obliterate the dead space in the pilonidal sinus, achieving quick recovery, was reported by Greenberg et al. [9]. Figure 2 demonstrates how fibrin glue can be applied to the pilonidal wound area. 

However, the evidence is not strong enough to assume the fibrin glue is an ideal treatment for pilonidal disease [10]. Therefore, it is essential to review the results of the recent clinical studies on fibrin glue and compare them to the previous ones to determine whether we can use it widely in multiple centers across the world. The study's objective was to judge the effect of fibrin glue as the fundamental treatment after having the sinus cleaned.

## Review

Method

Study Protocol

Our systematic review followed the Preferred Reporting Items for Systematic Reviews and Meta-Analyses (PRISMA) 2020 guidance [11]. 

Sources of Data Collection and Search Strategy

Information was searched from PubMed®, Ovid EMBASE®, Ovid MEDLINE®, and Cochrane CENTRAL. The last date of the search was the 10th of May 2021. Keywords and Medical Subject Heading (MeSH) were combined with Boolean operators during the search in these four databases. The full search strategy used in the PubMed include: ("Fibrin Tissue Adhesive"[MeSH Terms] OR ("fibrin"[MeSH Terms] OR "fibrin"[All Fields] OR "fibrins"[All Fields] OR "fibrine"[All Fields]) OR ("fibrinogen"[MeSH Terms] OR "fibrinogen"[All Fields] OR "fibrinogens"[All Fields] OR "fibrinogen s"[All Fields] OR "fibrinogene"[All Fields]) OR ("Fibrin Tissue Adhesive"[MeSH Terms] OR ("fibrin"[All Fields] AND "tissue"[All Fields] AND "adhesive"[All Fields]) OR "Fibrin Tissue Adhesive"[All Fields] OR "tissucol"[All Fields]) OR ("calcium chloride"[MeSH Terms] OR ("calcium"[All Fields] AND "chloride"[All Fields]) OR "calcium chloride"[All Fields] OR "Fibrin Tissue Adhesive"[MeSH Terms] OR ("fibrin"[All Fields] AND "tissue"[All Fields] AND "adhesive"[All Fields]) OR "Fibrin Tissue Adhesive"[All Fields] OR "tisseel"[All Fields] OR "fibrinogen"[MeSH Terms] OR "fibrinogen"[All Fields] OR "fibrinogens"[All Fields] OR "fibrinogen s"[All Fields] OR "fibrinogene"[All Fields] OR "fibrinolysis inhibitor"[All Fields] OR "thrombin human"[All Fields] OR "thrombin"[MeSH Terms] OR "thrombin"[All Fields] OR "thrombin s"[All Fields] OR "thrombine"[All Fields] OR "thrombins"[All Fields]) OR ("Fibrin Tissue Adhesive"[MeSH Terms] OR ("fibrin"[All Fields] AND "tissue"[All Fields] AND "adhesive"[All Fields]) OR "Fibrin Tissue Adhesive"[All Fields] OR "tissel"[All Fields]) OR ("Fibrin Tissue Adhesive"[MeSH Terms] OR ("fibrin"[All Fields] AND "tissue"[All Fields] AND "adhesive"[All Fields]) OR "Fibrin Tissue Adhesive"[All Fields] OR "beriplast"[All Fields]) OR ("Fibrin Tissue Adhesive"[MeSH Terms] OR ("fibrin"[All Fields] AND "tissue"[All Fields] AND "adhesive"[All Fields]) OR "Fibrin Tissue Adhesive"[All Fields] OR "crosseal"[All Fields]) OR ("Fibrin Tissue Adhesive"[MeSH Terms] OR ("fibrin"[All Fields] AND "tissue"[All Fields] AND "adhesive"[All Fields]) OR "Fibrin Tissue Adhesive"[All Fields] OR "transglutine"[All Fields])) AND ("Pilonidal Sinus"[MeSH Terms] OR ("pilonidal"[All Fields] AND ("abscess"[MeSH Terms] OR "abscess"[All Fields] OR "abscesses"[All Fields] OR "abscessation"[All Fields] OR "abscessed"[All Fields] OR "abscessing"[All Fields])) OR ("Pilonidal Sinus"[MeSH Terms] OR ("pilonidal"[All Fields] AND "sinus"[All Fields]) OR "Pilonidal Sinus"[All Fields] OR ("pilonidal"[All Fields] AND "cyst"[All Fields]) OR "pilonidal cyst"[All Fields]) OR ("pilonidal"[All Fields] AND ("disease"[MeSH Terms] OR "disease"[All Fields] OR "diseases"[All Fields] OR "disease s"[All Fields] OR "diseased"[All Fields]))). Table 1 represents each database, search strategy and result. 

**Table 1:** Database, search strategy, and result

Database	Search Strategy	Result
PubMed	Full strategy as above	28
Ovid Medline	Pilonidal sinus and fibrin tissue adhesive	26
Ovid Embase	Pilonidal sinus and fibrin tissue adhesive	60
Cochrane Central	Pilonidal sinus and fibrin tissue adhesive	12

Eligibility Criteria

Both new and recurrent pilonidal sinus disease were considered in this study. We included articles that reported the outcome of fibrin glue as the primary treatment with the cleaning of the sinus. There was no limitation to the age of patients. Any brand of fibrin sealant, including fibrinogen or a mixture of fibrinogen and thrombin, was studied. The inclusion criteria were articles published within the last ten years (2011-2021), studies related to humans only, and those published in English. We excluded review articles, those published earlier than 2011, and non-English papers. 

Study Selection Process

Two authors independently identified records via databases, followed by the removal of duplicates. Titles and abstracts of studies were then screened. We excluded non-relevant documents. Reports of relevant themes were retrieved to assess their eligibility. We applied the eligibility criteria mentioned above to include great studies in our review. The opinion from the third author was sought when the agreement was not achieved between the two authors throughout the selection process. We also designed a data extraction form to collect the data from the included studies.

Risk of Bias and Quality Assessment

We included six observational studies assessed for bias using the Newcastle-Ottawa Scale (NOS). Two reviewers independently judged the risk of bias in each article. In addition, a discussion with the third author was performed for any variability in the risk of inclination between the two reviewers. 

Results

**Figure 3:**
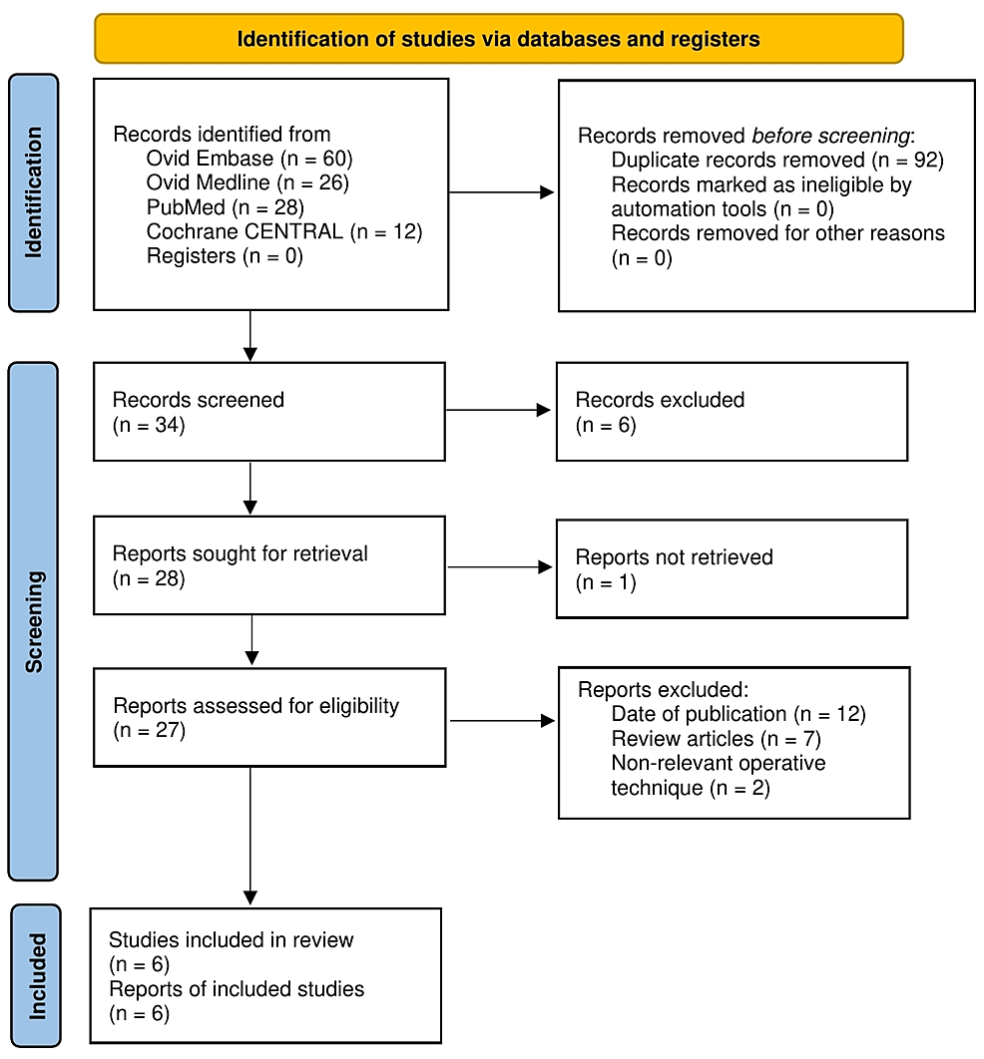
PRISMA flow diagram PRISMA: Preferred Reporting Items for Systematic Reviews and Meta-Analyses [11]

One hundred and twenty-six articles were retrieved from the searching of databases. Ninety-two duplicates were removed, leaving 34 papers. Six themes were non-relevant after screening the abstracts and titles; therefore, they were excluded from the review. Out of 28 reports, one report could not be recovered. We were able to retrieve 27 articles to be assessed for eligibility. With the careful consideration of inclusion and exclusion criteria, two authors independently assessed the remaining articles and selected six articles finally. This process can be seen in the PRISMA flow diagram, Figure 3. 

We extracted the data from selected six articles. The demographics such as the author's name, year of publication, location of study, sample size, age, and the number of male patients are presented in Table 2. 

**Table 2:** Demographics of the studies PC: primary closure, FS: fibrin sealant

No	References	Year	Country	Sample size	Male	Age
1	Hardy et al. [12]	2019	UK	18	10	16 (15-17)
2	Sian et al. [13]	2018	UK	146	115	28 (16-78)
3	Saedon et al. [14]	2018	UK	PC – 17, FS – 17	PC – 12, FS – 15	PC – 30 ± 15, FS – 29 ± 12
4	Smith et al. [15]	2015	UK	41	22	15 (12-16)
5	Isik et al. [16]	2014	Turkey	40	32	24 (16-50)
6	Elsey et al. [17]	2012	UK	57	42	26 (17-70)

Table 3 demonstrates categories, duration of follow-up, and interpretation of these studies.

**Table 3:** Characteristics of the included studies FGO: fibrin glue obliteration, PSD: pilonidal sinus disease, PC: primary closure, FS: fibrin sealant, PEF: pit excision and fibrin glue, FG: fibrin glue

References	Study Type	Treatment group	Control group	Follow up (months)	Conclusion
Hardy et al. [12]	Retrospective study	Curettage of tract + fibrin glue	_	13	FGO produced a good outcome with a 95% of success rate after a single application.
Sian et al. [13]	Retrospective study	Curettage of tract + fibrin glue	_	12	Fibrin glue application was a fast and productive treatment as both the first and second-line treatment of PSD.
Saedon et al. [14]	Comparative study	Cleaning of sinus tract + fibrin sealant	Sinus tract excision + primary closure	PC – 5, FS – 4	A selected group of patients benefited from the fibrin glue treatment with an excellent comparable outcome.
Smith et al. [15]	Comparative study	Pit excision + fibrin sealant	Lateralizing flap procedure	32	PEF was recommended for both primary and recurrent pilonidal disease in children.
Isik et al. [16]	Retrospective study	Curettage of tract + fibrin glue	_	18	Fibrin glue was advised as the first-line treatment in simple pilonidal sinus disease with a single non-infected sinus.
Elsey et al. [17]	Cross-sectional study	Curettage of sinus + fibrin glue	_	_	Patients were delighted with the fibrin glue treatment, and FG facilitated a quick return to normal activities. It was suggested as a first-line treatment for most of the pilonidal sinuses.

Discussion

The management of the pilonidal disease can be divided into operative and non-operative management [18]. Despite the various strategies to treat the condition, postprocedural complications and vicious cycle repetition can develop due to multiple factors, including gender, obesity, smoking, substandard personal care, and surgical techniques [19]. 

Post-Procedural Infection

Almost all of our included studies reported the rate of infection developed after the intervention. Smith et al. reported that the group treated with pit excision and fibrin glue (PEF) had a comparable infection rate managed by lateralizing flap procedure (LFP). The infection rate was seven percent in the group treated with LFP, whereas eight percent in the PEF group. Forty-one children with a mean age of 15 were included in this study. The sealant they injected was TISSEEL (Baxter Healthcare, Deerfield, Illinois) [15].

The comparative study by Saedon et al. evaluated the outcome of those who had cleaning the sinus tract obliterated by fibrin sealant with the other group who had excision of the sinus tract and primary closure. The sample size was 34, with an equal division between the two groups. Those managed with fibrin sealant revealed an infection rate of six percent, while those allocated to have excision and subsequent closure resulted in a 47% postoperative infection rate. There was an outstanding contrast in the infection rate among the groups with a p-value of 0.045. But this evidence was uncertain since the antibiotic was given before the procedure in some patients with infection [14].

Hardy et al. also reported a low rate of infection, which was 11.1%. It was a retrospective study of new and recurrent pilonidal sinus disease in those aged 15-17. It investigated the effect of fibrin glue, TISSEEL, applied in the cleaned sinus tract without giving any prophylactic antibiotic [12]. 

These studies described non-identical surgical procedures and the application of fibrin glue with diverse brands and volumes. Some of them comprised of small sample size, and none of them was a randomized controlled trial. Two studies focused on only pediatric participants. There was no uniformity in the judgment of the use of pre-operative antibiotics. Due to this variableness among studies, the overall impact of fibrin glue on the rate of infection is inconclusive. This finding is consistent with the previous systematic review conducted by Lund et al. [10]. 

Recurrence of Pilonidal Sinus

Return of the disease is one of the most critical outcomes of interest for both clinicians and patients. The condition is esteemed to have a recurrence if the clinical features reappear and at least one firm or two soft-recurrence criteria are met after the wound has fully healed without any injury to the natal cleft postoperatively [20]. Various factors such as the number and size of the pilonidal sinus, the immediate suturing of the wound after the sinus are removed, and the family history is the risk factors for the return of the disease [21]. 

Every surgical treatment for the pilonidal disease has its recurrence rate, which varies depending on the type of surgical technique and the duration of the follow-up. For example, a low recurrence rate of 0.6% was reported in Limberg & Dufourmentel operations whereas 0.2% in Karydakis & Bascom procedures at 12-month follow-up period. However, primary midline closure was associated with a recurrence rate up to 67.9% 20 years after surgery [22]. Furthermore, according to our included studies, the recurrence rate after fibrin glue therapy with cleaning the sinus tract ranges from 5.6% to 29% [12-16]. 

Isik et al., one of these retrospective studies, assessed patients who had a new presentation of a single sinus without previous infection or treatment. They looked into 40 cases. Fibrin glue was used to obliterate the dead space left after the sinus tract was curetted. It reported a recurrence rate of 10% [16]. Sian et al. was also a similar retrospective study. However, a more significant number of patients, 146 in total, and complex cases with multiple natal cleft pits were reviewed. A recurrence rate of 27% was reported by this study [13].

The different range of follow-up and the nature of the pilonidal sinus in participants could be the possible causes of this variability among the studies. The follow-up ranged from four months to 32 months. Generally, the recurrence after fibrin glue therapy does not indicate concern according to the results of included studies. 

Return to Normal Activities

Since the pilonidal disease manifests mainly in the productive age group, the time taken to restart work and school is a significant concern from the socio-economic point of view. Therefore, this factor becomes one of the outcomes of interest. Hardy et al. found that the median time for daily tasks was only three days [12]. Elsey et al. also received the responses from the patients that it took a week generally to resume everyday actions [17]. Our studies highlighted that more minor procedures were associated with a quicker return to activities.

Patient Satisfaction

Elsey et al. was the only study that discussed patients' satisfaction rate after having the fibrin glue procedure. This cross-sectional study evaluated the responses of 57 patients who had undergone cleaning of the sinus tract with a surgical scoop followed by fibrin glue injection. Cases were carefully selected, excluding those with acute abscesses and multiple recurrences. 79% of patients were pleased with the outcome due to rapid recovery, less surgical stress, and less pain after receiving this management [17]. The ability to offer this satisfaction is also one of the crucial points in considering the ideal treatment.

Why Our Study I/s Important

Various therapeutic techniques with multiple studies are available to treat the pilonidal disease, which means the ideal one is not recognized yet. According to previously published review articles, the quality of evidence is not strong enough to confirm the impact of fibrin glue on managing pilonidal disease [10, 23]. However, additional clinical studies have been designed and published after previous systematic reviews to explore fibrin glue. It is vital to keep looking into the best solution for the disease, which carries significant morbidity to the productive age group. Therefore, we carried out our study focusing on more recent studies to reflect on the up-to-date science. Our review will be helpful in consideration of the role of fibrin glue in pilonidal management.

Limitations of Our Study

Our systematic review analyzed six publications, including the recent studies. However, some limitations exist, such as excluding unpublished studies and having no randomized controlled trial to review. Moreover, we had only studies between 2011 and 2021 to focus on the updated information. As a result, the review might not be comprehensive.

## Conclusions

Our review is to evaluate the therapeutic effect of fibrin glue on pilonidal disease. Although our studies report relatively better outcomes regarding the recurrence rate, infection, and the time to return to normal activities, more extensive studies with proper randomization and longer duration of follow-up are still essential to confirm this weak promising result of fibrin glue. Moreover, none of these studies discussed its cost related to its primary and recurrent therapy. Therefore, the success rate compared to its price is debatable. Since other cost-effective procedures with comparable results are available, future studies should look into them meticulously. It will help discover the most feasible and ideal therapeutic option for pilonidal disease. Further research should also provide more robust evidence for using fibrin glue to fill the sinus tract under local anesthesia in the ambulatory service or general practice among selected patients.
